# Single-shot T1 mapping of the corpus callosum: a rapid characterization of fiber bundle anatomy

**DOI:** 10.3389/fnana.2015.00057

**Published:** 2015-05-11

**Authors:** Sabine Hofer, Xiaoqing Wang, Volkert Roeloffs, Jens Frahm

**Affiliations:** ^1^Biomedizinische NMR Forschungs GmbH, Max-Planck-Institut für Biophysikalische ChemieGöttingen, Germany; ^2^Bernstein Center for Computational NeuroscienceGöttingen, Germany

**Keywords:** MRI, human brain, T1 mapping, corpus callosum, fiber anatomy

## Abstract

Using diffusion-tensor magnetic resonance imaging and fiber tractography the topographic organization of the human corpus callosum (CC) has been described to comprise five segments with fibers projecting into prefrontal (I), premotor and supplementary motor (II), primary motor (III), and primary sensory areas (IV), as well as into parietal, temporal, and occipital cortical areas (V). In order to more rapidly characterize the underlying anatomy of these segments, this study used a novel single-shot T1 mapping method to quantitatively determine T1 relaxation times in the human CC. A region-of-interest analysis revealed a tendency for the lowest T1 relaxation times in the genu and the highest T1 relaxation times in the somatomotor region of the CC. This observation separates regions dominated by myelinated fibers with large diameters (somatomotor area) from densely packed smaller axonal bundles (genu) with less myelin. The results indicate that characteristic T1 relaxation times in callosal profiles provide an additional means to monitor differences in fiber anatomy, fiber density, and gray matter in respective neocortical areas. In conclusion, rapid T1 mapping allows for a characterization of the axonal architecture in an individual CC in less than 10 s. The approach emerges as a valuable means for studying neocortical brain anatomy with possible implications for the diagnosis of neurodegenerative processes.

## Introduction

The corpus callosum (CC) is a unique structure in placental mammals and by far the largest fiber bundle in the human brain interconnecting the two cerebral hemispheres with more than 300 million fibers (de Lacoste et al., 1985; Clarke and Zaidel, 1994; Aboitiz and Montiel, 2003). In humans and other primates the CC features a rough topographical representation of different cortical areas, in which anterior cortical areas are connected through the anterior CC and posterior areas through posterior regions (Aboitiz et al., 1992; Hofer and Frahm, 2006; Hofer et al., 2008; van der Knaap and van der Ham, 2011). The CC has been the target of extensive *in vivo* studies (e.g., see Thompson et al., 2003) indicating that its morphology may be related to a large variety of disorders such as dyslexia (von Plessen et al., 2002), depression (Lacerda et al., 2005; Cyprien et al., 2014), schizophrenia (Narr et al., 2002), HIV/AIDS (Thompson et al., 2006), autism (Prigge et al., 2013), and neonatal motor function (Mathew et al., 2013).

Although the CC can be identified by conventional magnetic resonance imaging (MRI), there are no *in vivo* anatomic landmarks that clearly delimit distinct callosal areas in a midsagittal cross-section (van der Knaap and van der Ham, 2011). Using diffusion tensor imaging and fiber tractography, Hofer and Frahm (2006) distinguished five major segments of the CC, containing fibers projecting into prefrontal (region I), premotor and supplementary motor (region II), primary motor (region III), and primary sensory areas (region IV), as well as into parietal, temporal, and occipital cortical areas (region V). The functional specialization of these segments most likely results from well-defined pathways of interhemispheric communication where characteristic transfer properties are based on fiber composition. The density of thin fibers is most apparent in the anterior CC (genu, region I), with fiber diameters between 0.4 and 1 μm. The fiber density decreases to a minimum in the somatomotor region (region III–IV; fiber diameter mostly between 2 and 7 μm), and increases again toward the posterior CC (splenium, region V), with a mixed population of fiber diameters. Unmyelinated fibers were found to be scarce, except in the genu where they comprised about 16% of total fibers (Aboitiz et al., 1992).

Qualitative tissue contrast of anatomic MR images is based on differences in the density of water protons and their relaxation times. To exploit these relationships in order to obtain more reliable information about the tissue microstructure, it is necessary to detail the contributions of different contrast mechanisms such as given by the relaxation times T1, T2, and T2*. In fact, quantitative parametric MRI studies have been of increasing interest in recent years. For example, the longitudinal relaxation time T1 describes the recovery of magnetization from a perturbed state to its equilibrium state. It mainly reflects the mobility of water protons. Accordingly, quantitative T1 evaluations have been shown to enhance the pathological specificity by an improved differentiation of healthy and affected tissue within individual subjects. Anatomic variations between “normal appearing white matter,” “diffusely abnormal white matter” and discernable white matter lesions were the topic of numerous clinical studies (Dreha-Kulaczewski et al., 2009; Hagemeyer et al., 2012; West et al., 2014). In multiple sclerosis, increased water T1 values are linked to increased water content, caused by edema, and increased extracellular space, caused by extracellular loss and demyelination (Vrenken et al., 2010).

In general, cerebral white matter often contains distinct regions with either small unmyelinated fibers or large and gigantic myelinated fibers. These differences in microstructure lead to regional variations in water content and extracellular space and in turn affect the respective T1 relaxation times. Or conversely, T1 relaxation times of (normal) white matter reflect the fiber anatomy and underlying cellular composition. This work therefore applied a novel rapid T1 mapping method (Wang et al., 2015) to study the CC in healthy human subjects where the type and distribution of axonal fibers are well-known. To properly cover callosal portions with densely packed small fiber bundles and gigantic fibers with large extracellular space, regions-of-interest for a quantitative T1 evaluation were placed according to the CC parcellation scheme of Hofer and Frahm (2006).

## Materials and Methods

Ten healthy male subjects (age range 18–35 years) participated in this study. Written informed consent, according to the recommendations of the local ethics committee, was obtained from all subjects prior to MRI. MRI studies were conducted at 3 T (Magnetom Prisma, Siemens Healthcare Erlangen, Germany) using a 64-channel head coil. Anatomic images were based on a T1-weighted 3D fast low angle shot (FLASH) MRI sequence (repetition time TR = 11 ms, echo time TE = 4.9 ms, flip angle 15°).

Single-shot T1 mapping of the CC was performed at 0.75 mm in-plane resolution and 6 mm section thickness in a midsagittal position without involving the lateral and third ventricles as well as the surrounding gray matter. T1 mapping was based on a single-shot inversion-recovery experiment (Look and Locker, 1970; Deichmann and Haase, 1992) with radial undersampling (25 spokes, TR = 3.29 ms, TE = 2.00 ms, flip angle 4°) at a temporal resolution of 82.25 ms per frame. The total acquisition time was 8 s and measurements were repeated three times.

The time course of the MRI signal can pixel-wise be described by

$$M(t)=M0^{*}-(M0^{*}+M0)\;\exp\left(-t/{T1}^{*}\right)$$

with M0^∗^ the observed steady-state magnetization, M0 the equilibrium magnetization, and 1/T1^∗^ = 1/T1–1/TR log(cos(α)). Assuming TR < < T1^∗^,T1, the desired T1 value can be calculated (Deichmann and Haase, 1992) according to

$$T1={T1}^{*}M0/M0^{*}$$

Serial image reconstruction was performed in reversed chronological order based on regularized non-linear inversion (NLINV) for parallel MRI (Uecker et al., 2008) which was extended to real-time MRI at high temporal resolution (Uecker et al., 2010, 2012). The latter version was adapted to reconstruct the image series obtained by the inversion-recovery experiment prior to pixel-wise fitting (Wang et al., 2015).

**FIGURE 1:**
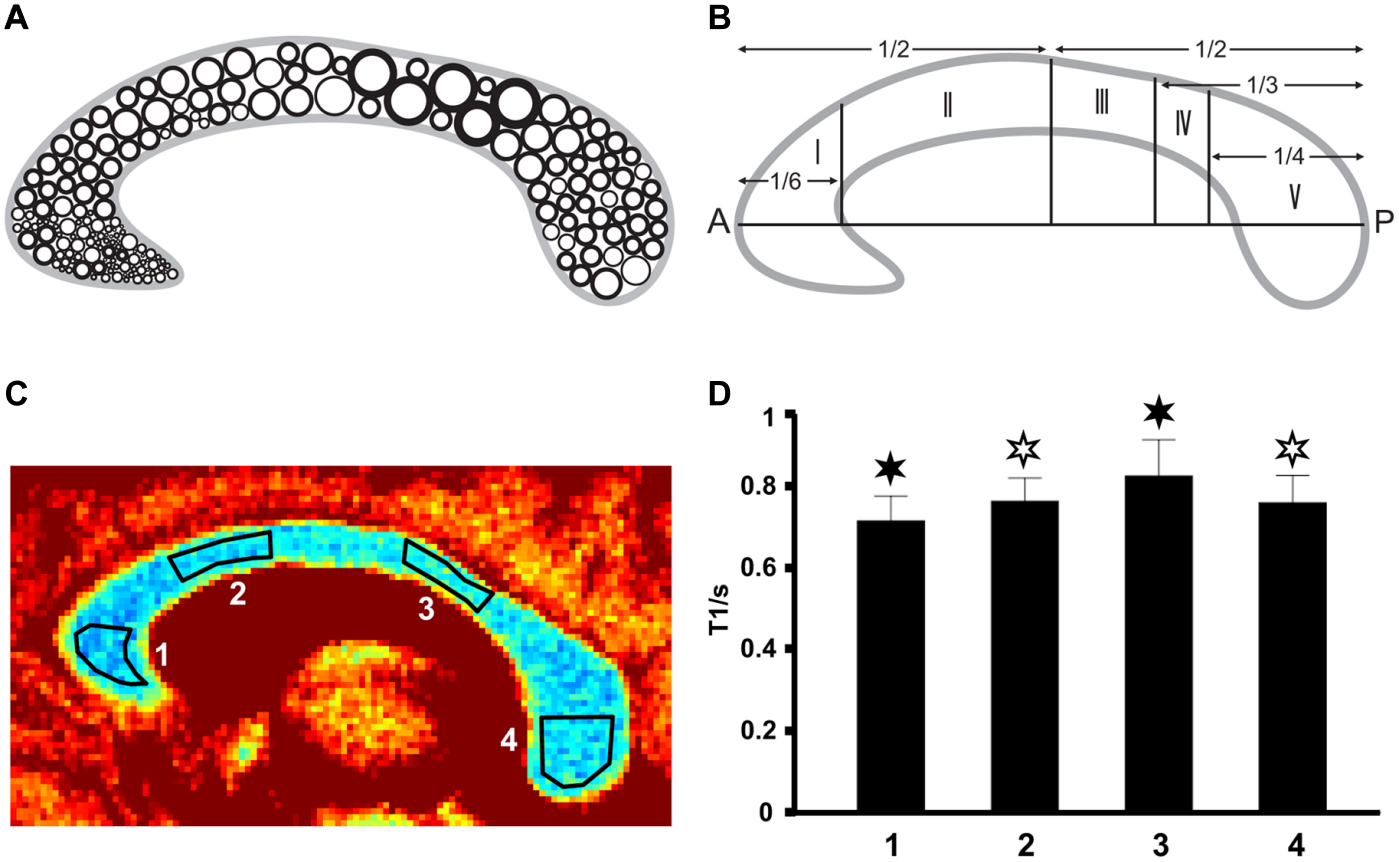
**(A)** Fiber composition in the midsagittal corpus callosum (CC; modified after Aboitiz et al., 1992) with large circles representing thick fibers and small circles representing thin fibers. **(B)** Geometric parcellation of the CC (Hofer and Frahm, 2006) with I = prefrontal, II = premotor and supplementary motor, III = primary motor, IV = primary somatosensory, and V = parietal, temporal, occipital. (C) Color-coded T1 map of the CC (subject VII) with ROI definitions: low T1 values are in blue and high T1 values appear in red. (D) Corresponding mean T1 relaxation times in the four regions (subject VII). Filled asterisks indicate significant differences to all other areas, open asterisks indicate significant differences to other areas except to the second area marked with an open asterisk.

To cover specific subregions of the CC, we used the geometric parcellation scheme described by Hofer and Frahm (2006). ROIs were manually drawn on grayscale maps and placed within the genu (region I = 1), the anterior midbody (region II = 2), the somatomotor region (region III–IV = 3) as well as the splenium (region V = 4) as indicated in **Figures 1A–C**. Data analysis, reconstruction, and ROI definition were performed using MATLAB 2013a (MathWorks, Natick, MA, USA). Regional differences of mean T1 values were tested for significance (Superior Performing Software System, SPSS Inc.) using ANOVA combined with a *post hoc* test (Bonferroni) for multiple comparisons at a threshold of *p* < 0.05. Analyses were performed between different CC regions in every individual as well as in a group analysis.

## Results

The three repetitive measurements in each subject resulted in very similar T1 values with no statistically significant differences (not shown here). Because of this high intra-subject reproducibility, the subsequent results only used the first data set for each subject. We further found the single-shot measurements for T1 mapping to be virtually free of motion artifacts. Moreover, in contrast to other quantitative MRI studies, we evaluated absolute T1 relaxation times across all subjects without individual normalization (Hofer and Frahm, 2006; Hofer et al., 2008).

**FIGURE 2:**
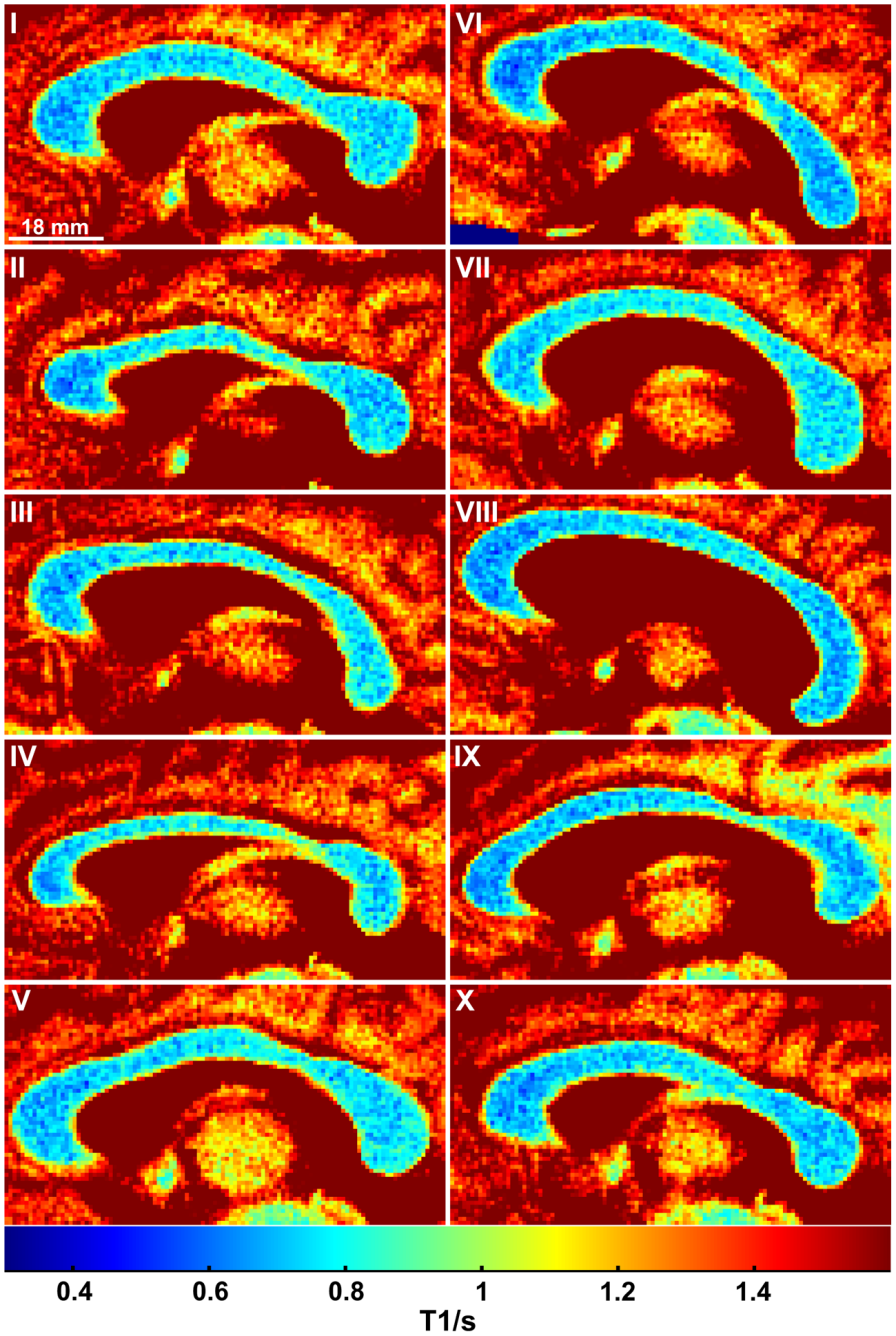
**Color-coded T1 maps of the CC for all 10 subjects**.

As shown in **Figure 1C** (color-coded T1 map) and **Figure 1D** (regional distribution of T1 values) for a single subject, the T1 relaxation times in the most anterior part of the CC (genu) were significantly lower compared to the anterior midbody, somatomotor region, and splenium. Only subject IX presented with significantly lower values in the genu compared to the somatomotor and splenium region, but no significant different values to the anterior midbody. The color-coded T1 maps for all 10 subjects are shown in **Figure 2**, while quantitative T1 values and statistically significant differences are summarized in **Table 1**. The T1 relaxation times in the somatomotor region were in most subjects significantly higher compared to all other CC regions; significance only failed in subject VIII for the anterior midbody. In contrast, the T1 relaxation times of the anterior midbody and splenium with its mixed fiber population were not significantly different to each other in individual subjects in almost all cases.

**Table 1:** T1 relaxation times (ms, mean ± SD) in the corpus callosum and significant regional differences (*p* < 0.05).

Subject	1 Genu	2 Anterior midbody	3 Somatomotor	4 Splenium
I	691 ± 6	734 ± 6	804 ± 7	743 ± 6
II	671 ± 7	761 ± 7	861 ± 8	734 ± 7
III	695 ± 7	756 ± 7	838 ± 10	777 ± 8
IV	689 ± 7	771 ± 6	874 ± 8	747 ± 8
V	703 ± 6	739 ± 6	800 ± 7	770 ± 6
VI	649 ± 8	714 ± 7	768 ± 8	693 ± 6
VII	716 ± 6	764 ± 6	827 ± 8	760 ± 7
VIII	680 ± 7	718 ± 6	752 ± 7	712 ± 7
IX	679 ± 7	705 ± 6	823 ± 9	729 ± 8
X	684 ± 6	719 ± 6	778 ± 7	739 ± 6
I	2,3,4	1,3	1,2,4	1,3
II	2,3,4	1,3	1,2,4	1,3
III	2,3,4	1,3	1,2,4	1,3
IV	2,3,4	1,3	1,2,4	1,3
V	2,3,4	1,3,4	1,2,4	1,2,3
VI	2,3,4	1,3	1,2,4	1,3
VII	2,3,4	1,3	1,2,4	1,3
VIII	2,3,4	1	1,4	1,3
IX	3,4	3	1,2,4	1,3
X	2,3,4	1,3	1,2,4	1,3

**FIGURE 3:**
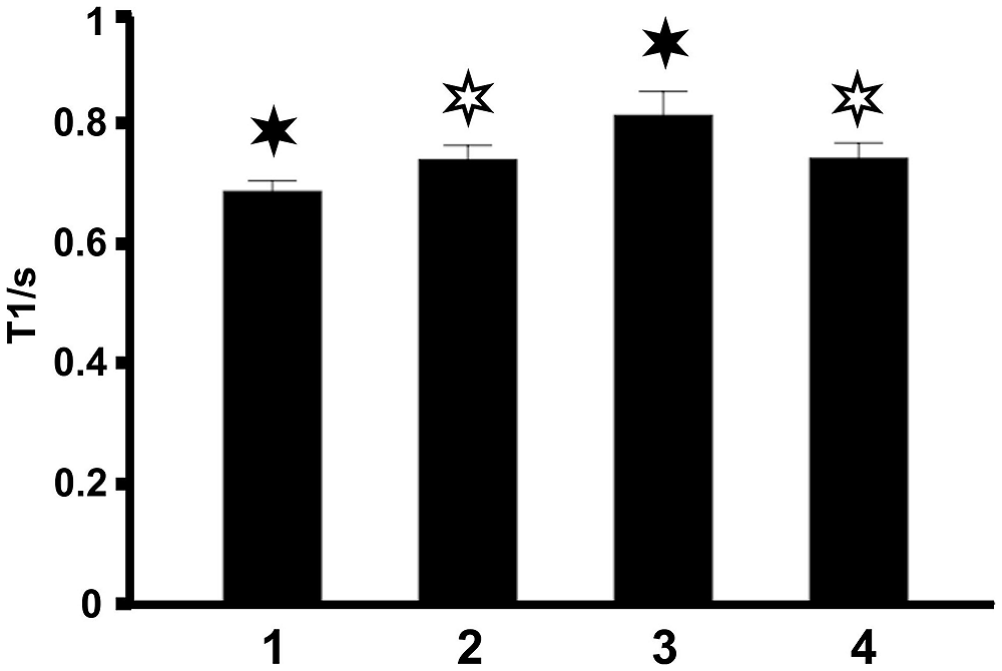
**Mean T1 relaxation times in the four regions of the CC averaged across all 10 subjects.** Filled asterisks indicate significant differences to all other areas, open asterisks indicate significant differences to other areas except to the second area marked with an open asterisk.

The same findings were obtained by taking the mean T1 relaxation times averaged across all 10 subjects (**Figure 3**). This observation demonstrates that T1 relaxation times in midsagittal CC sections are highly reproducible between individuals.

## Discussion

In this study, we found variations of T1 relaxation times in four different regions of the human CC, which are in line with the known fiber anatomy in respective topographic locations. Our data show a trend for higher T1 values in the somatomotor region with large fiber diameters and lower axon density. Accordingly, there is no common T1 relaxation time for the entire CC: the longitudinal relaxation mechanism directly reflects differences in water content and mobility which are associated with fiber anatomy and in turn depend on fiber thickness, degree of myelination, extracellular space, and fiber density.

In contrast to other white matter areas in the brain (e.g., in parietal, occipital, or frontal hemispheric areas) which are characterized by small u-fibers and large association and projection tracts including populations of gigantic myelinated and small unmyelinated fibers (Mori et al., 2002; Schmahmann and Pandya, 2006; Schmahmann et al., 2007), the CC consists of regions with distinct and well-described fiber populations (de Lacoste et al., 1985; Aboitiz et al., 1992; Clarke and Zaidel, 1994; Aboitiz and Montiel, 2003). These established anatomic features render it possible to define relations between *in vivo* T1 values and underlying white matter microstructure. In particular, the lowest T1 relaxation times were found in the genu of the CC with densely packed thin fibers with less myelin, whereas the highest T1 relaxation times were found in the somatomotor region characterized by bundles of gigantic fibers. In fact, increased water proton T1 relaxation times, as shown here in areas with gigantic fibers, are linked to increased water content and mobility caused by increased extracellular space (Vrenken et al., 2010). On the other hand, mixed fiber populations with large and small myelinated fibers as in the anterior midbody and parts of the splenium show similar T1 values, due to similar axonal densities and degrees of myelination.

A common problem for *in vivo* MRI studies of tissue microstructure is partial volume effects. In white matter partial volume effects are to be considered when different fibers or fiber bundles reside in a single image voxel. Here, the use of ROI analyses of specific CC regions in healthy subjects circumvented most of this problem and helped to establish a robust relationship between fiber composition and absolute T1 relaxation time. Moreover, overlapping signal contributions from white matter, gray matter, and cerebrospinal fluid could be minimized by choosing an adequate slice thickness to avoid any coverage of the ventricles as well as cingulate white and gray matter. Finally, high-resolution mapping of the midsagittal fiber profile of the CC was ensured by using 0.75 mm in-plane resolution, while the novel rapid single-shot method (Wang et al., 2015) not only minimized putative motion artifacts, but also resulted in highly reproducible T1 values in each subject within a measuring time of seconds.

The current finding of T1 differences in relation to CC fiber anatomy is in line with previous results obtained by other techniques (Hofer and Frahm, 2006; Hofer et al., 2008; Horowitz et al., 2014) and further agrees with postmortem findings (Aboitiz et al., 1992). A recent diffusion MRI study (Horowitz et al., 2014) revealed a similar distribution of fiber diameters in the CC as originally demonstrated by electron microscopy (Aboitiz et al., 1992): axon diameters are narrow in the genu, broad in the body part, and moderate in the splenium. However, the diffusion-weighted echo planar imaging method required a long acquisition time of about 10 min and also resulted in image distortions (Horowitz et al., 2014; Innocenti et al., 2015).

Seewann et al. (2009) showed in a combined postmortem MRI and histopathological study of the brains of patients suffering from multiple sclerosis that T1 relaxation times in diffuse abnormal white matter are correlated with diffusion metrics such as fractional anisotropy, apparent diffusion coefficient and axonal counts, as well as with axonal and myelin density. Higher T1 relaxation times in brain tissue were reported to correlate with reduced myelin density, increased fibrillary gliosis, inflammatory brain regions, or axonal loss (Seewann et al., 2009; Vrenken et al., 2010).

In comparison to previous studies (Hofer and Frahm, 2006; Hofer et al., 2008), the T1 mapping proposed here appears to be the fastest and most sensitive neuroanatomical mapping technique *in vivo*, with the potential for interindividual regional comparisons without the need for value normalization. Furthermore, the high experimental accuracy provides great flexibility, and will allow for the monitoring of disease progression and brain development or plasticity processes in individual subjects.

Absolute T1 relaxometry with access to microstructural tissue properties may find manifold clinical applications. For example, this may in particular apply to abnormal white matter in chronic multiple sclerosis with special emphasis on the CC as an indicator for neocortical atrophy.

## Conflict of Interest Statement

The authors declare that the research was conducted in the absence of any commercial or financial relationships that could be construed as a potential conflict of interest.

## References

[B1] Aboitiz F. Montiel J. (2003). One hundred million years of interhemispheric communication: the history of the corpus callosum. *Braz. J. Med. Biol. Res.* 36 409–420 10.1590/S0100-879X2003000400002 12700818

[B2] Aboitiz F. Scheibel A. B. Fisher R. S. Zaidel E. (1992). Fiber composition of the human corpus callosum. *Brain Res.* 598 143–153 10.1016/0006-8993(92)90178-C 1486477

[B3] Clarke J. M. Zaidel E. (1994). Anatomical-behavioral relationships: corpus callosum morphometry and hemispheric specialization. *Behav. Brain Res.* 64 185–202 10.1016/0166-4328(94)90131-7 7840886

[B4] Cyprien F. Courtet P. Poulain V. Maller J. Meslin C. Bonafé A. (2014). Corpus callosum size may predict late-life depression in women: a 10-year follow-up study. *J. Affect. Disord.* 165 16–23 10.1016/j.jad.2014.04.040 24882172

[B5] Deichmann R. Haase A. (1992). Quantification of T1 values by Snapshot-FLASH NMR imaging. *J. Magn. Reson.* 96 608–612 10.1016/0022-2364(92)90347-A

[B6] de Lacoste M. C. Kirkpatrick J. B. Ross E. D. (1985). Topography of the human corpus callosum. *J. Neuropathol. Exp. Neurol.* 44 578–591 10.1097/00005072-198511000-00004 4056827

[B7] Dreha-Kulaczewski S. F. Helms G. Dechent P. Hofer S. Gärtner J. Frahm J. (2009). Serial proton MR spectroscopy and diffusion tensor imaging in infantile Balo’s concentric sclerosis. *Neuroradiology* 51 113–121 10.1007/s00234-008-0470-y 18958461 PMC2726919

[B8] Hagemeyer N. Goebbels S. Papiol S. Kästner A. Hofer S. Begemann M. (2012). A myelin gene causative of a catatonia-depression syndrome upon aging. *EMBO Mol. Med.* 4 528–539 10.1002/emmm.201200230 22473874 PMC3443947

[B9] Hofer S. Frahm J. (2006). Topography of the human corpus callosum revisited - comprehensive fiber tractography using diffusion tensor magnetic resonance imaging. *Neuroimage* 32 989–994 10.1016/j.neuroimage.2006.05.044 16854598

[B10] Hofer S. Merboldt K. D. Tammer R. Frahm J. (2008). Rhesus monkey and human share a similar topography of the corpus callosum as revealed by diffusion tensor MRI in vivo. *Cereb. Cortex* 18 1079–1084 10.1093/cercor/bhm141 17709556

[B11] Horowitz A. Barazany D. Tavor I. Bernstein M. Yovel G. Assaf Y. (2014). In vivo correlation between axon diameter and conduction velocity in the human brain. *Brain Struct. Funct.* 220 1789–1790 10.1007/s00429-014-0871-0 25139624

[B12] Innocenti G. M. Caminiti R. Aboitiz F. (2015). Comments on the paper by Horowitz et al. (2014). *Brain Struct. Funct.* 220 1777–1788 10.1007/s00429-014-0974-7 25579065

[B13] Lacerda A. L. Brambilla P. Sassi R. B. Nicoletti M. A. Mallinger A. G. Frank E. (2005). Anatomical MRI study of corpus callosum in unipolar depression. *J. Psychiatr. Res.* 39 347–354 10.1016/j.jpsychires.2004.10.004 15804385

[B14] Look D. Locker D. (1970). Time saving in measurement of NMR and EPR relaxation times. *Rev. Sci. Instrum.* 41 250–251 10.1063/1.1684482

[B15] Mathew P. Pannek K. Snow P. D’Acunto M. G. Guzzetta A. Rose S. E. (2013). Maturation of corpus callosum anterior midbody is associated with neonatal motor function in eight preterm-born infants. *Neural Plast.* 2013:359532 10.1155/2013/359532 PMC3569930 23509639

[B16] Mori S. Kaufmann W. E. Davatzikos C. Stieltjes B. Amodei L. Fredericksen K. (2002). Imaging cortical association tracts in the human brain using diffusion-tensor-based axonal tracking. *Magn. Reson. Med.* 47 215–223 10.1002/mrm.10074 11810663

[B17] Narr K. L. Cannon T. D. Woods R. P. Thompson P. M. Kim S. Asunction D. (2002). Genetic contributions to altered callosal morphology in schizophrenia. *J. Neurosci.* 22 3720–3729. 11978848 10.1523/JNEUROSCI.22-09-03720.2002 PMC6758392

[B18] Prigge M. B. Lange N. Bigler E. D. Merkley T. L. Neeley E. S. Abildskov T. J. (2013). Corpus callosum area in children and adults with autism. *Res. Autism Spectr. Disord.* 7 221–234 10.1016/j.rasd.2012.09.007 23130086 PMC3487714

[B19] Schmahmann J. D. Pandya D. N. (2006). *Fiber Pathways of the Brain*. Oxford: Oxford University Press. 10.1093/acprof:oso/9780195104233.001.0001

[B20] Schmahmann J. D. Pandya D. N. Wang R. Dai G. D’Arceuil H. E. de Crespigny A. J. (2007). Association fibre pathways of the brain: parallel observations from diffusion spectrum imaging and autoradiography. *Brain* 130 630–653 10.1093/brain/awl359 17293361

[B21] Seewann A. Vrenken H. van der Valk P. Blezer E. L. Knol D. L. Castelijns J. A. (2009). Diffusely abnormal white matter in chronic multiple sclerosis: imaging and histopathologic analysis. *Arch. Neurol.* 66 601–609 10.1001/archneurol.2009.57 19433660

[B22] Thompson P. M. Dutton R. A. Hayashi K. M. Lu A. Lee S. E. Lee J. Y. (2006). 3D mapping of ventricular and corpus callosum abnormalities in HIV/AIDS. *Neuroimage* 15 12–23 10.1016/j.neuroimage.2005.11.043 16427319

[B23] Thompson P. M. Narr K. L. Blanton R. E. Toga A. W. (2003). “Mapping structural alterations of the corpus callosum during brain development and degeneration,” in *The Parallel Brain: The Cognitive Neuroscience of the Corpus Callosum* eds Zaidel E. Iacoboni M. (Cambridge, MA: MIT Press) 93–130.

[B24] Uecker M. Hohage T. Block K. T. Frahm J. (2008). Image reconstruction by regularized nonlinear inversion – joint estimation of coil sensitivities and image content. *Magn. Reson. Med.* 60 674–682 10.1002/mrm.21691 18683237

[B25] Uecker M. Zhang S. Voit D. Karaus A. Merboldt K. D. Frahm J. (2010). Real-time MRI at a resolution of 20 ms. *NMR Biomed.* 23 986–994 10.1002/nbm.1585 20799371

[B26] Uecker M. Zhang S. Voit D. Merboldt K. D. Frahm J. (2012). Real-time MRI – recent advances using radial FLASH. *Imaging Med.* 4 461–476 10.2217/iim.12.32

[B27] van der Knaap L. J. van der Ham I. J. (2011). How does the corpus callosum mediate interhemispheric transfer? A review. *Behav. Brain Res.* 30 211–221 10.1016/j.bbr.2011.04.018 21530590

[B28] von Plessen K. Lundervold A. Duta N. Heiervang E. Klauschen F. Smievoll A. I. (2002). Less developed corpus callosum in dyslexic subjects – a structural MRI study. *Neuropsychologia* 40 1035–1044 10.1016/S0028-3932(01)00143-9 11900755

[B29] Vrenken H. Seewann A. Knol D. L. Polman C. H. Barkhof F. Geurts J. J. (2010). Diffusely abnormal white matter in progressive multiple sclerosis: in vivo quantitative MR imaging characterization and comparison between disease types. *Am. J. Neuroradiol.* 31 541–548 10.3174/ajnr.A1839 19850760 PMC7963986

[B30] Wang X. Roeloffs V. Merboldt K. D. Voit D. Schätz S. Frahm J. (2015). Single-shot multi-slice T1 mapping at high spatial resolution – inversion-recovery FLASH with radial undersampling and iterative reconstruction. *Open Med. Imaging J.* 9 1–8 10.2174/1874347101509010001

[B31] West J. Aalto A. Tisell A. Leinhard O. D. Landtblom A. M. Smedby Ö. (2014). Normal appearing and diffusely abnormal white matter in patients with multiple sclerosis assessed with quantitative MR. *PLoS ONE* 9:e95161 10.1371/journal.pone.0095161 PMC3991609 24747946

